# The Crosstalk Between Tumor Cells and the Immune Microenvironment in Breast Cancer: Implications for Immunotherapy

**DOI:** 10.3389/fonc.2021.610303

**Published:** 2021-03-11

**Authors:** Vincenzo Salemme, Giorgia Centonze, Federica Cavallo, Paola Defilippi, Laura Conti

**Affiliations:** Department of Molecular Biotechnology and Health Sciences, University of Torino, Torino, Italy

**Keywords:** breast cancer, cancer immunotherapy, tumor microenvironment (TME), immune checkpoint inhibitors (ICI), immunosuppression

## Abstract

Breast cancer progression is a complex process controlled by genetic and epigenetic factors that coordinate the crosstalk between tumor cells and the components of tumor microenvironment (TME). Among those, the immune cells play a dual role during cancer onset and progression, as they can protect from tumor progression by killing immunogenic neoplastic cells, but in the meanwhile can also shape tumor immunogenicity, contributing to tumor escape. The complex interplay between cancer and the immune TME influences the outcome of immunotherapy and of many other anti-cancer therapies. Herein, we present an updated view of the pro- and anti-tumor activities of the main immune cell populations present in breast TME, such as T and NK cells, myeloid cells, innate lymphoid cells, mast cells and eosinophils, and of the underlying cytokine-, cell–cell contact- and microvesicle-based mechanisms. Moreover, current and novel therapeutic options that can revert the immunosuppressive activity of breast TME will be discussed. To this end, clinical trials assessing the efficacy of CAR-T and CAR-NK cells, cancer vaccination, immunogenic cell death-inducing chemotherapy, DNA methyl transferase and histone deacetylase inhibitors, cytokines or their inhibitors and other immunotherapies in breast cancer patients will be reviewed. The knowledge of the complex interplay that elapses between tumor and immune cells, and of the experimental therapies targeting it, would help to develop new combination treatments able to overcome tumor immune evasion mechanisms and optimize clinical benefit of current immunotherapies.

## Introduction

Breast cancer (BC) still represents the most frequent cancer in women and the second cause of cancer deaths worldwide (1). Treatment options have improved the outcome of BC patients, but still many patients progress to metastatic disease, which remains very difficult to cure. The failure of specific therapies may be ascribed to the fact that most anti-cancer drugs currently used mainly target cancer cells. Indeed, emerging evidence suggests that BC is not only composed of neoplastic cells but also of the tumor microenvironment (TME) consisting of different cell types, including endothelial cells, several stromal cell types, and immune cells. The cells composing the TME undergo a complex interplay with cancer cells through either cell–cell contacts or the production of extracellular matrix complexes and soluble factors that shape the microenvironment (2). The continuous and dynamic interaction between cancer cells and the TME can either promote or hinder cancer progression. In particular, tumor infiltrating immune cells protect from tumor progression by eliminating immunogenic neoplastic cells, but in the meanwhile they can contribute to tumor resistance to therapies, shaping tumor immunogenicity and selecting resistant tumor clones able to escape the immune response (3). Although BC was previously considered as a poor immunogenic cancer that does not respond to immunotherapies due to a low mutational burden (4), the notion of the role exerted by the immune system in BC progression has led to the application of this type of treatments also in this tumor. The introduction of immunotherapies improved the outcome of many BC patients, however, data from the clinics have underlined that it is strongly influenced by the composition of the immune TME. Indeed, immune cells have been implied in the development of resistance mechanisms to immunotherapy in BC, which hampers the establishment of durable responses, leading to disease progression (5).

Therefore, a deeper knowledge of BC TME and of the role that the different tumor infiltrating immune cell populations exert on cancer progression and response to therapies would allow the development of more effective treatments for BC. Furthermore, the identification of TME-related characteristics associated with a good or poor response to therapies would facilitate patient stratification and therapeutic decisions. In this light, in this paper we summarize the role exerted by the main immune cell populations present in the TME in BC progression, their influence on immunotherapies, and we discuss novel therapeutic strategies able to counteract the tumor-promoting activities of BC TME.

## Major Players in BC Immune Microenvironment

**Figure 1:**
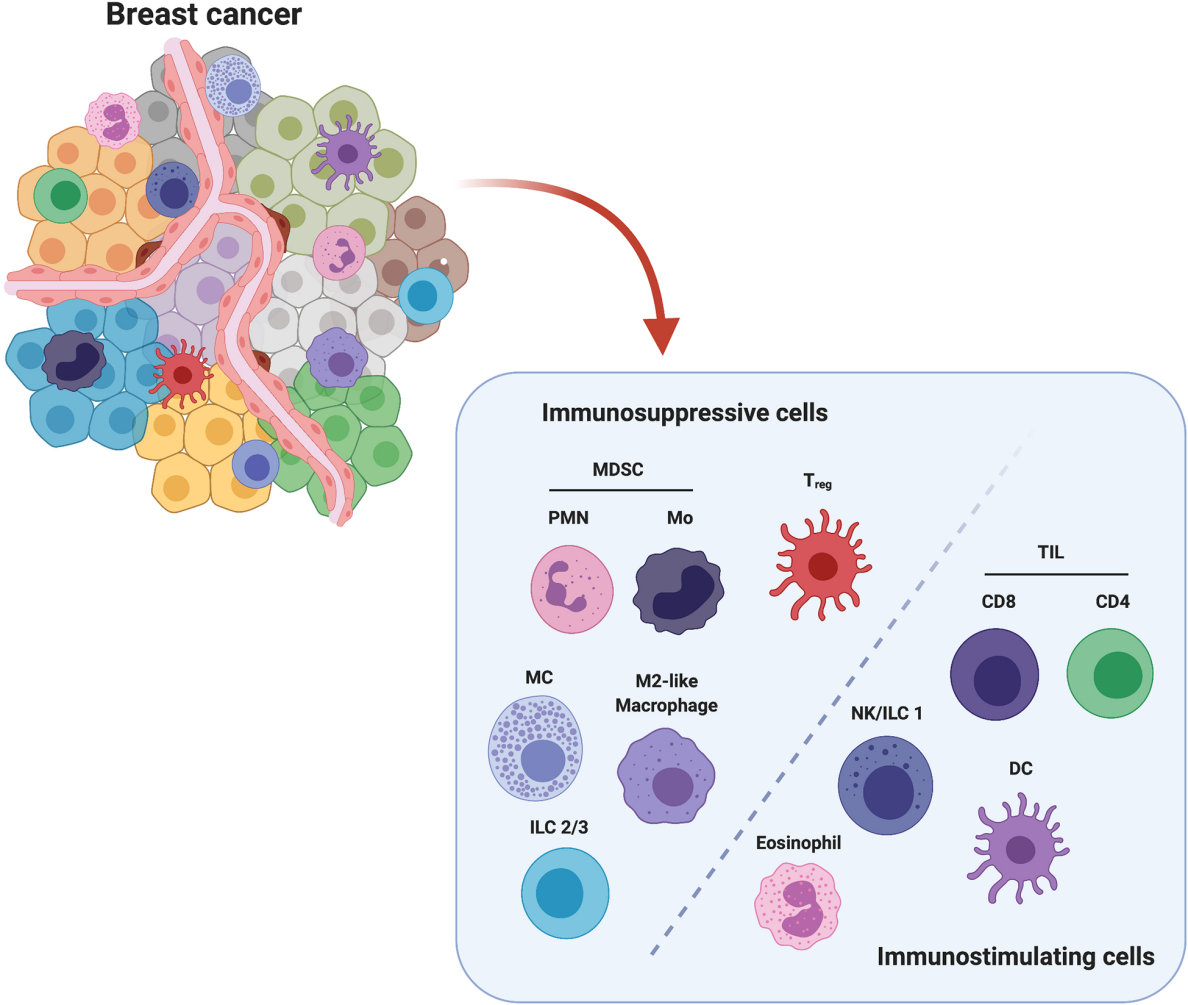
Major players in immune breast TME. Among all cell populations present in breast TME, polymorphonuclear (PMN) and monocytic (Mo) Myeloid-Derived Suppressor Cells (MDSCs), Mast Cells (MCs), Innate Lymphoid Cells Type 2 and 3 (ILC2/3), M2-like Tumor Associated Macrophages (TAMs) and FoxP3^+^ regulatory T cells (Treg) are considered to exert an immunosuppressive action, while Tumor Infiltrating CD4^+^ and CD8^+^ Lymphocytes (TILs), Natural killer (NK) cells/Innate Lymphoid Cells Type 1 (ILC1), Dendritic cells (DCs) and Eosinophils are associated with an anti-tumor activity. Created with BioRender.com.

During the evolutionary history of a tumor, a complex and dynamic communication between tumor cells and the cells in the TME is established, shaping several tumor hallmarks such as sustained proliferative signaling, avoidance of immune destruction, induction of angiogenesis, and activation of invasion and metastasis (6). Importantly, different types of immune cells play specific roles, establishing a strong crosstalk network with cancer cells (**Figure 1**). In this sense, tumor immunoediting by innate and adaptive immune cell populations that together constitute the so-called Breast Cancer Immune Microenvironment (BCIM) is an important determinant of tumor progression. Immunoediting is a dynamic process that occurs in three steps, notably Elimination, Equilibrium, and Escape. The Elimination is the first step, also called immunosurveillance, in which transformed cells are destroyed by a competent immune system able to activate a strong immune response against cancer. During the Equilibrium phase, tumor cells that survived the Elimination phase and immune cells reciprocally shape each other. A balance is established between the tumor and the immune system with a selection pressure on tumor cells, which are genetically unstable and rapidly mutating. Tumor cell variants that have acquired resistance to elimination then enter the Escape phase, the final step of the process, when the tumor grows and becomes clinically apparent. The Escape phase is characterized by the progressive establishment of an immunosuppressive TME (7).

Based on the activity of the innate and adaptive immune cell populations involved in the immunoediting process, we can identify two major subclasses of immune cells: the immunosuppressive and the immunostimulating cells. Several lines of evidence have demonstrated that the presence of these cells within the BCIM significantly impacts on BC progression and treatment response. In particular, infiltration of tumors by immunostimulating immune cells such as some macrophages, lymphocytes, natural killer (NK) cells, innate lymphoid cells (ILCs), dendritic cells (DCs) and eosinophils is crucial for tumor control (8). The anti-cancer immune response generated by these cells is, however, inhibited by the action of immunosuppressive cells, such as myeloid-derived suppressor cells (MDSCs), mast cells (MCs), regulatory T cells (Tregs), and type 2-polarized tumor-associated macrophages (M2-like TAMs), which are intrinsically associated with the developing TME (9).

Here, we briefly describe the major immune subpopulations present in BCIM, with a particular attention to their impact on BC patient’s prognosis and to their influence on the response to current immunotherapies. In addition, we review the state of the art of the therapeutic strategies aiming at reverting immunosuppression in order to potentiate anti-cancer immune responses.

### Immunosuppressive Cells

#### Myeloid-Derived Suppressor Cells

MDSCs are a heterogeneous population of progenitors and precursors of myeloid cells. The molecular mechanisms behind their generation and their true origins are still debated, and different theories proposed. Upon an increased demand for myeloid cells, immature myeloid cells (IMCs) can undergo a process known as emergency myelopoiesis, expanding in the bone marrow and migrating into the periphery. Or else, IMCs may also expand and become functionally active MDSCs extramedullary (in organs such as spleen) (10). Conversely, in pathologic conditions such as cancer, several cytokines, chemokines and factors, such as for example granulocytic-colony stimulating factor (G-CSF) (11), C-X-C-chemokine ligand (CXCL)2, CC-chemokine ligand (CCL)2, CCL5 (12) CXCL5, and CXCL12 (13) (see below *Cytokine and Soluble Factors-Mediated Mechanisms*) secreted by the tumor cause the block of their differentiation as well as their mobilization from the bone marrow and accumulation into the primary and secondary neoplastic lesions (10). Based on the different cell surface antigen expressions, two subsets of MDSCs have been identified: polymorphonuclear or granulocytic MDSCs (PMN-MDSCs) and monocytic MDSCs (M-MDSCs). In mice, the PMN-MDSCs and M-MDSCs are identified by a CD11b^+^Ly6G^+^Ly6C^low^ and a CD11b^+^Ly6G^−^Ly6C^high^ phenotype, respectively, whereas, in humans, PMN-MDSCs are CD11b^+^CD14^−^CD15^+^CD33^+^ cells, and M-MDSCs are CD11b^+^CD14^+^ CD15^−^CD33^+^HLA^−^DR^−/low^ cells. Other hypotheses suggest that M-MDSCs and PMN-MDSCs may represent reprogrammed or activated monocytes and granulocytes (10). Nowadays, it is widely accepted that these IMCs, through the secretion of several soluble factors as well as the production of reactive oxygen species (ROS) and reactive nitrogen species (RNS) (see below), are able to induce severe anergy of effector immune cells, to recruit Tregs and to promote the M2-like TAM polarization, thus generating a strong immunosuppressive TME. In particular, MDSCs are able to recruit Tregs at the tumor site throughout the expression on their membrane of the immune stimulatory receptor CD40. The same receptor is exploited by MDSCs to directly inhibit T-cell proliferation by its binding with the ligand CD40L expressed on T-cell plasma membrane (14, 15). Recently, MDSCs have also been associated with the formation of the pre-metastatic niche, to the stimulation of angiogenesis and the maintenance of cancer stem cells (CSCs), a small population of cells responsible for tumor initiation and metastases (16–18). Several studies have shown that MDSCs are associated with poor prognosis in BC patients. Notably, Kumar et al. reported that MDSCs are more enriched in triple-negative BC (TNBC) patient samples compared to non-TNBC (19), and high levels of circulating MDSCs significantly correlate to liver and bone metastases and higher levels of circulating tumor cells (20). In summary, many lines of evidence suggest that MDSCs play a detrimental role in BC progression.

#### Mast Cells

MCs are innate immune cells characterized by their cargo of inflammatory mediators stored in cytoplasmic granules, which are released upon encountering the appropriate stimuli, such as IgE, that play a central role in allergic diseases (21). MC degranulation is known to have beneficial roles in response against pathogens, such as helminths, bacteria, and viruses.

They are distributed in diverse tissues throughout the body and, like other immune cells, originate into the bone marrow from the hematopoietic stem cell progenitor which can become a committed MC progenitor that through the bloodstream migrates to peripheral tissues to complete maturation (22). Their differentiation, growth, and survival are strongly regulated by tissue microenvironmental factors, of which stem cell factor (SCF), the ligand of the c-Kit receptor, and interleukin (IL)-3 are the best-characterized (23).

Interestingly, other endogenous factors such as IL-4, IL-6, IL-9, IL-10, IL-33, nerve growth factor (NGF), and transforming growth factor *β* (TGF-*β*) contribute to MC maturation and function (22). Inside the tumor, MCs are able to suppress the anti-tumor immune response by inducing an adenosine-mediated immunosuppressive crosstalk with MDSCs and Tregs and by limiting the adaptive immunity through IL-13 secretion (24, 25). However, the influence of MCs in BC prognosis is still much debated. MCs, through the secretion of the great variety of bioactive components contained inside the cytoplasmic granules, may exert both pro- and anti-tumor effects. In particular, *in vitro* and *in vivo* studies indicate that MCs exhibit a pro-tumor activity through the promotion of lymphatic and blood vessel formation, tumor growth, and metastasis (26). On the other hand, Samoszuk et al. demonstrated that depletion of MCs with imatinib enhanced tumor growth in a murine model of BC, supporting MC anti-tumoral role (27). Another study associates MCs with a greater survival and favorable prognosis (28). Consistently, Rajput et al. reported that in a cohort of 4.444 invasive BC patients with a long term follow-up, stromal MCs correlate with a good prognosis (29).

#### M2-Like Tumor Associated Macrophages

Macrophages are terminally differentiated myeloid cells which are responsible for the elimination of infectious agents and the regulation of adaptive immunity. For many years, macrophage biological origin was attributed to bone marrow-derived progenitors and blood monocyte intermediates that differentiate into mature cells once seeded into organs (30). However, several genetic tracing data revealed that multiple macrophage populations develop from embryonic progenitors and are able to self-renew by local proliferation of mature, differentiated cells. Each tissue microenvironment has been demonstrated to influence macrophage morphological and functional characteristics (31). Based on their functional role, macrophages have been classified in two different subtypes: anti-tumoral M1-like and pro-tumoral M2-like polarized TAMs (32). In mice, both M1- and M2-like TAMs are characterized by the expression of markers such as CD11b, F4/80 and colony-stimulating factor-1 receptor (CSF-1R) and low levels of expression of the myeloid differentiation marker Gr1, whereas major histocompatibility complex (MHC) class II glycoproteins and CD206 are used to distinguish between M1- and M2-like TAMs, respectively. In humans, macrophages are identified by the expression of CD68, CD312, CD115, and other markers. However, it is important to note that TAM phenotypes are much more complex and categorizing them into binary states is not completely correct (33). Several data indicate that the pro-tumoral M2-like TAMs within the BCIM play pivotal roles in promoting tumorigenesis and metastasis formation *via* both non-immune and immune related mechanisms. The non-immune role of TAMs consists in the release of numerous angiogenic factors, such as vascular endothelial growth factor (VEGF), platelet-derived growth factor (PDGF), and basic fibroblast growth factor (bFGF), that stimulate angiogenesis within the tumor, as well as in the secretion of many signaling molecules, including EGF, matix metalloproteinases (MMPs), CCL2, CCL18, and macrophage (M)-CSF that consequently activate tumor cell epithelial–mesenchymal transition (EMT), invasion, and metastasis (34, 35). The pro-tumoral M2-like TAM infiltration contributes to establish an immunosuppressive microenvironment. For example, it has been reported that M2-like TAMs, through the secretion of TGF-*β*, as well as IL-10, suppress CD8^+^ T cell functions by direct transcriptional repression of genes encoding functional mediators, such as perforins, granzymes, and cytotoxins (34, 36). Moreover, in virtue of their high expression levels of enzymes such as arginase 1 (ARG1) and indoleamine 2,3-dioxygenase 1, M2 TAMs deplete the TME of the amino acids arginine and tryptophan, which are essential for T and NK cell proliferation and survival (35) (see below). Several studies demonstrated that M2-TAMs are a poor prognostic factor in BC (37–39). In particular, M2-TAMs promote tumor growth by facilitating immunosuppression, angiogenesis, and inflammation, and can also promote tumor recurrence after conventional therapies (30, 39). Consistently, CSF1-expressing TAMs are associated with more aggressive tumors, in a cohort of 47 BC patients (33). Moreover, signatures of M2-like TAM infiltration correlate with a poor prognosis in luminal and triple negative subgroups of BC (40).

#### FoxP3^+^ Regulatory T Cells

Tregs are a distinct specialized subpopulation of T cells that act to suppress immune response. Tregs represent half of the CD4^+^CD25^+^ T cell population. In addition, a small number of CD8^+^FoxP3^+^ Tregs have also been identified in a large cohort of BC patients (41). Physiologically, Tregs are involved in the regulation of T and B lymphocyte activation as well as in the homeostasis of cytotoxic lymphocytes (9, 42). The normal thymus produces FoxP3-expressing CD25^+^CD4^+^ Tregs. In addition to these naturally occurring Tregs, some naive CD25^–^CD4^+^ T cells may also differentiate to Tregs in the periphery (43). Tregs are also involved in a broad spectrum of pathologies such as autoimmunity, allograft rejection, and hypersensitivity. Their role in immunosuppression is indisputable since they can disrupt the host immune response through a multitude of mechanisms involving cell–cell contacts and the production of immunosuppressive cytokines and metabolites, thus sustaining tumor progression and aggressiveness. Tregs appear to have a major role in disrupting the immune control of cancers and are therefore associated with worse patient outcome (44).

Higher numbers of Tregs in the peripheral blood of BC patients compared with healthy controls have been reported, and their ability to infiltrate tumors increases with tumor stage and correlates with poor prognosis in invasive BCs (41, 45). Tregs are recruited in the TME by several chemokines and cytokines produced by tumor cells, cancer associated fibroblasts or immunosuppressive cells. CXCL12 is one of the main factors that induce Treg recruitment. Interestingly, the expression of CXCL12 and its receptor CXCR4 is increased by hypoxia, which could further promote Treg infiltration in breast tumors, especially in the basal-like subtype (46). Related to the different BC subtypes, it has been described that Treg infiltration signature is associated with poor prognosis in luminal, triple negative and HER2^+^ BC. Interestingly, Peng et al. also reported that, in a cohort of 122 patients with primary invasive ductal BC, patients with a low FoxP3^+^/CD8^+^ ratio showed a higher disease free survival (DFS) than patients with an higher FoxP3^+^/CD8^+^ ratio (47). Moreover, the depletion of Tregs in advanced primary tumors induces a strong CD4^+^ T cell and interferon (IFN)*γ*-dependent anti-tumor response (45). In particular, the interferon (IFN) *γ* derived from the CD4^+^ cells, but not from the CD8^+^ and NK cells, is responsible for the tumoricidal effects after Treg depletion in PyMT breast carcinomas (48).

### Anti-Tumor Immune Cells

#### Tumor Infiltrating T cells

TILs include all the cells with a lymphocytic nature infiltrating the tumor tissues. Of particular interest are cytotoxic (CD8^+^) and helper (CD4^+^) T-lymphocytes (49) that constitute an essential part of the adaptive immunity. CD8^+^ T-lymphocytes are the major effector cells involved in tumor elimination by recognizing tumor-associated- and neo-antigens presented by MHC class I (47). CD4^+^ T cells can support and help the CD8^+^ T population during the anti-tumor response *via* the secretion of a wide range of effector cytokines. In general, TIL abundance in tumors is fundamental for the establishment of an important immune response against cancer. Indeed, a huge literature is consistent with a positive correlation between TILs and good prognosis of BC patients. For example, an increased number of TILs positively correlates with increased DFS and overall survival (OS) in both TNBC and HER2-positive BC patients treated with neoadjuvant chemotherapy. Surprisingly, this correlation is completely lost in luminal A tumors (50). Further studies are needed to elucidate the underlying mechanisms, which might be related to the effects of the endocrine therapy on the immune system in Luminal A patients.

#### Natural Killer Cells

NK cells derive from a common lymphoid progenitor into the bone marrow and then spread to primary and secondary lymphoid tissues, as well as within non-lymphoid tissues including the lungs, liver, and the peripheral blood (51, 52). Phenotypically, they are identified as CD3^−^NK1.1^+^ in mice, while in humans two main subsets exist: cytotoxic CD56^dim^CD16^+^ cells and cytokine-producing CD56^bright^CD16^−^ cells (51). In both mice and humans, NK cells can be divided in four subsets, corresponding to different maturation stages, based on the expression of CD27 and CD11b surface markers. Immature NK cells do not express the two markers. During maturation, they acquire CD27 expression and then CD11b, while fully mature NK present in peripheral blood are nearly all CD11b^+^ CD27^−^. These different phenotypes correspond to different cell functions, with CD27^+^ cells showing the best ability to secrete cytokines, and CD11b^+^ CD27^−^ displaying high cytolytic function (53, 54). NK cells play an important role in cancer immunosurveillance, eliminating a variety of transformed cells through the release of cytolytic granules containing perforins and granzymes. Differently from T-lymphocytes, NK cells participate in the innate immunity and can recognize and kill altered cells without prior sensitization. Moreover, NK cells recognize and eliminate cells that do not express MHC class I, a mechanism that many cancer cells, and BC CSCs in particular, exploit to escape from T cell-mediated cytotoxicity (55, 56). For these reasons, NK cells are the most effective immune cell subpopulation to control and eventually eliminate abnormal cells. However, in BC and several other solid cancer types, tumor infiltrating NK cells display a CD56^bright^CD16^−^ phenotype and secrete invasion-associated enzymes such as MMP9 and, similarly to decidual NK cells, exert pro-angiogenic functions through the secretion of VEGF and angiogenin (57, 58). VEGF induces tumor vessel growth and exerts immunosuppressive functions, promoting the proliferation of immunosuppressive cells, limiting T-cell recruitment and enhancing T-cell exhaustion (59). This shift in NK cell function may be induced by several factors present in the breast TME, as previously described for lung cancer, where TGF-*β*, adenosine, and prostaglandins downregulate NK activating receptors and induce the production of VEGF and placental growth factor (PIGF) that promote cancer progression (55, 60, 61). Interestingly, the balance between pro- and anti-tumor activity exerted by NK cells differs in the different BC subtypes. Indeed, a strong presence of NK cells that in turn is associated with a good prognosis has been found in ER^+^ and HER2^+^ BC patients, while NK cell infiltration correlates with poor prognosis in TNBC patients (40).

#### Innate Lymphoid Cells

ILCs are immune cells deriving from the common lymphoid progenitor and belong to the innate counterparts of T cells. In effect, ILCs have been proposed as the evolutionary precursors of T cells that do not express antigen-specific receptors (62). They are tissue resident cells extremely rare in the peripheral blood (63, 64), able to detect changes in the local microenvironment through receptors for cytokines that are released during tissue damage, and to trigger the adaptive immunity (65). Based on their hallmarks, such as their cytokine signature and phenotype, ILCs are divided into three major groups: ILC1s, ILC2s, and ILC3s, even if two additional immune cell types, NK cells and lymphoid tissue inducer cells, are also included in the ILC family (66).

In response to IL-12, IL-15, and IL-18, ILC1s secrete IFN*γ* that is extremely important to induce macrophages and DCs to eliminate bacteria and to present antigens. ILC2s secrete type-2 cytokines such as IL-5, IL-9, IL-13, and amphiregulin, which on one hand are involved in the expulsion of helminths and in helping to repair the damaged tissues, while on the other hand are able to enhance Treg functions and thus immunosuppression (67). ILC3s, instead, produce IL-22 and IL-17 that are able to stimulate the secretion of antimicrobial peptides and mucus by epithelial and goblet cells, respectively (68, 69).

Like NK cells, ILC1s are dependent on IL-15 and exhibit potent cytotoxic activities against tumor cells, limiting tumor growth in mammary preclinical model (70, 71). In BC, Irshad et al. identified an interesting mechanism through which ILC3s, together with stromal cells, are able to promote lymphatic metastasis by modulating the local chemokine milieu. In particular, in a preclinical mouse model of TNBC, CCL21-dependent ILC3 recruitment into the primary tumor stimulates CXCL13 production by the stromal cells, which in turn promotes the production of the cancer cell motile factor RANKL that induces cell migration (72). Moreover, in BC an enrichment of ILC2s in tumors compared to healthy tissue was observed, and IL-33 administration in 4T1 BC cell model accelerates tumor growth and the development of lung and liver metastases, which is associated with increased intratumoral infiltration of ILCs, MDSCs and Tregs (73, 74). However, the real contribution of ILCs in cancer disease is still a matter of debate. Whether the enrichment of ILCs into the tumor site results from newly recruited cells or from local *in situ* proliferation is another open question.

#### Dendritic Cells

DCs are specialized antigen-presenting cells able to orchestrate an efficient anti-tumor immunity as well as to participate in the immune tolerance. Mouse and human conventional DCs derive from common DC precursors in the bone marrow. There are two main subsets of DCs, monocytic DCs (mDCs) that are generally CD11c^+^, and plasmacytoid DCs (pDCs) (75, 76). DCs induce an efficient T lymphocyte activation and anti-tumor immune response stimulation through the process of antigen presentation on MHC class I and II molecules to T-lymphocytes, as well as by producing immunomodulatory signals through cell–cell contacts and soluble factors (77).

DCs have been found in many cancer types, including BC, where they are poorly activated and often dysfunctional, since the TME promotes their production of IL-10 and TGF-*β*, which contribute to the expansion of Tregs (77). Moreover, an increase of DCs has been observed in the peripheral blood of BC patients, with higher levels in HER2-positive BC patients compared to HER2 negative ones, suggesting differences between the different BC subtypes (78). However, the prognostic role of DCs in patients remains unclear, likely due to their heterogeneous composition that comprises cells at different maturation stages. In a recent study about metastatic BC, Holsbø and Olsen analyzed gene expression profiles in patient blood samples and examined genes and gene sets associated with risk of BC metastasis. Among the top genes, pDC-related genes and processes were identified (79). This was in line with another study, in which pDC infiltration in primary localized BC correlates with an adverse outcome, suggesting their contribution in tumor progression (80). On the other hand, Bailur and colleagues’ results suggest a positive association between circulating pDCs and BC survival (76, 80). Similarly, the presence of CD83^+^ mature intratumor DCs strongly associated with better patient survival in node-positive tumors (81), and CD11c^+^ mDCs positively correlated with T cell infiltration and OS in TNBC patients (82). Moreover, different subsets of DCs can have different correlations with therapeutic response in BC patients. Indeed, a significant increase of DCs in the blood was noted in BC patients whose tumors showed a good pathological response following neoadjuvant capecitabine and docetaxel preceded by adriamycin and cyclophosphamide regimens. However the presence of a decreased amount of intratumoral CD1a^+^ DCs did not show any significant correlation with response to therapy, in both primary breast tumors and metastatic axillary lymph nodes (83, 84).

#### Eosinophils

Eosinophils are innate immune cells involved in the protective immune response of the host against helminthes (85), viral (86) and microbial pathogens (87). Human eosinophils derive from CD34^+^CD117^+^ pluripotent hematopoietic stem cells in the bone marrow, where they complete their maturation and subsequently enter into the bloodstream (88).

Phenotypically, eosinophils are characterized as CD11b^+^Gr-1^lo^F4/80^+^ cells. These markers are also found on macrophages, but eosinophils can be distinguished due to their high granularity, lack of expression of MHC-II and expression of the sialic acid-binding lectin Siglec-F (89).

Eosinophils are recruited from the blood into the sites of inflammation where, upon activation, they can release an array of inflammatory mediators such as for example cationic proteins (major basic protein (MBP), eosinophil cationic protein (ECP), eosinophil peroxide (EPX), and eosinophil-derived neurotoxin (EDN)) that are unique to eosinophils and are important in the defense against parasitic infections (90). Noteworthy, IL-5 together with IL-3 and GM-CSF, is crucial for supporting the maturation of human eosinophils in the bone marrow (91) and mediates their survival by NF-*κ*B-induced Bcl-xL, which inhibits apoptosis.

Evidence indicates the presence of eosinophils in the TME of several human hematological and solid tumors, including BC, even if the mechanisms responsible of the eosinophil infiltration into the tumors are not completely known (92–94). However, some data show that the high-mobility group box 1 protein (HMGB1), IL-1α, and IL-33 potentially trigger eosinophil recruitment (95). Moreover, macrophages and MCs can recruit eosinophil *via* the production of VEGFs (96, 97) and/or the release of histamine and prostaglandin D_2_ (PGD2) through the activation of the chemoattractant-homologous receptor expressed on Th2 cells (CRTH2) (98) and H4 receptor (99), respectively.

Into the TME, eosinophils influence other leukocytes, such as T cells, NK cells, DCs and macrophages. In particular, they are able to recruit and activate T cells through CXCL9, CXCL10, and CCL5, to attract NK cells by IL-6, IL-12, and CXCL10 production, and to induce M1 polarization (100). Therefore, the presence of eosinophils into the tumor or in bloodstream is a favorable prognostic factor for most cancers, although evidence for a pro-tumorigenic role for eosinophils is reported (101). In BC eosinophils appear to be anti-tumorigenic, enhancing the patients’ ability to respond against disease (102). In particular, Ownby et al. reported that BC patients with eosinophil counts of less than 55/mm^3^ had significantly higher risk of recurrent disease than patients who had normal or high levels of eosinophils (102). Moreover, a study on a cohort of 930 BC patients reported a benefit for relative eosinophil count (REC)-high *vs* REC-low in BC-specific survival and in time to treatment failure (93).

## Mechanisms of Immunosuppression in Breast TME

**Figure 2:**
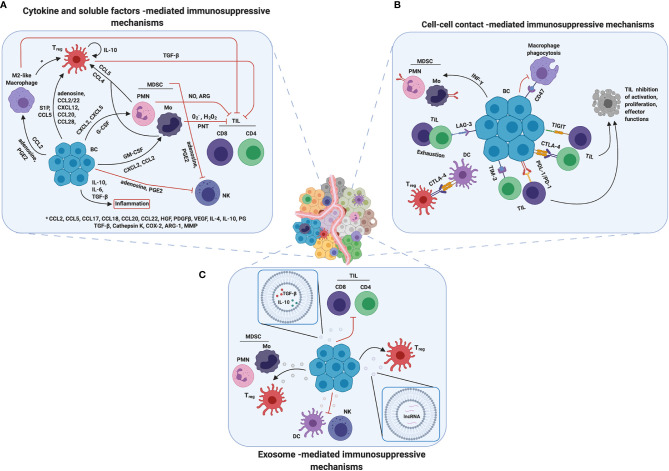
Mechanisms of immunosuppression in breast TME. Breast cancer cells developed several mechanisms to promote immunosuppression. **(A)** Cytokines and soluble factors are the main players in cell communication and signaling and they are able to mediate immune cell recruitment, mobilization and/or tumor infiltration. Moreover, they promote inflammation and contribute in changing TME composition, making it more immune suppressive. **(B)** Another strategy adopted by breast cancer cells is to overexpress on their surface immune checkpoint receptors such as PD-L1 or CTLA4, inducing cell–cell contact mediated death or anergy in T cells and suppressing immune response against tumor. **(C)** Finally, tumor derived exosomes could induce a reprograming in both immune suppressive and immune cells promoting tumor progression and survival by a wild range of molecules through different mechanisms. Created with BioRender.com.

During tumor progression, several immunosuppressive mechanisms appear, with a huge advantage in terms of growth, aggressiveness and resistance to treatments for cancer cells. As reported above, the BCIM contains specific immune sub-populations that, through complex and dynamic mechanisms, are able to inhibit the host anti-tumor immune response, by affecting the activity of the main immunostimulating populations. It is important to note that, to generate a tumor immunosuppressive microenvironment, the presence of the immunosuppressive cells inside the tumor lesion is absolutely indispensable. Several anti-inflammatory mechanisms used by BC cells to mobilize and recruit the immunosuppressive mediators have been identified. Here we summarize the main communication strategies that the tumor cells apply to recruit these pro-tumor immune cells as well as the mechanisms through which these cells inhibit the activity of the anti-tumor immune cells, distinguishing between cytokine/soluble factors, cell–cell contact and exosome-mediated mechanisms (**Figure 2**).

### Cytokine and Soluble Factors-Mediated Mechanisms

Colony-stimulating factors (CSFs) are essential for the proliferation, activity and differentiation of the myeloid-cell lineage. G-, GM- and M-CSF are the main components of this family. Interestingly, BC cells can upregulate the expression of these CSFs through a variety of mechanisms, promoting the mobilization and infiltration of specific MDSC populations into the tumors (12, 103, 104). In particular, the mTOR pathway drives G-CSF expression in *in vivo* preclinical models of BC, where, notably, the CSC compartment exhibits an elevated production of G-CSF, thus identifying a positive correlation between CSCs and immunosuppressive TME (11). In addition, it has been demonstrated that tumors actively reprogram metabolic pathways to evade effective anti-tumor immunity. Interestingly, a high glycolytic rate is associated with an increased secretion of both G-CSF and GM-CSF in TNBC cells (105).

Equally, also the chemokines play an important and fundamental role in the regulation of the TME. In particular, the secretion of CXCL2 and CCL22 by ΔNp63-carrying BC cells has been reported to be associated with MDSC infiltration. Importantly, CCL2 and CCL5 have been identified to be important chemokines implicated in monocyte and/or M-MDSC migration to tumors (12). Instead, CXCL5 and CXCL12 (SDF-1) play an important role in PMN-MDSC recruitment into the primary tumor in a BC mouse model with the deletion of *Tgfbr2* (13).

Once recruited inside the tumor, the MDSCs explicate a strong immunosuppressive activity both directly, through the continuous production of reactive oxygen species (ROS), nitric oxide (NO) and several cytokines and, indirectly, by attracting additional immunosuppressive populations. In particular, it is widely reported that M-MDSCs are able to produce mainly O_2_^–^, H_2_O_2_, and peroxynitrite (PNT), while PMN-MDSCs mainly release NO and arginase, which deplete L-arginine from the TME, inhibiting T cell function. These MDSC-derived ROS, NRS, and PNT are able to modify the T cell receptor (TCR) and the CD8 molecules, inducing the block of T-cell immune activity (106). Interestingly, MDSCs can directly block the entry of CD8^+^ T cells into tumors, by producing high levels of PNT, as well as are able to inhibit T-cell proliferation, strongly impairing the anti-tumor immune response (12, 107). MDSCs as well as the BC cells themselves can also produce immunosuppressive cytokines, such as IL-10, IL-6 and TGF-*β*, inducing inflammation that may facilitate immune suppression (108, 109). Moreover, to amplify the immunosuppression mechanisms repertoire, MDSCs are able to attract Tregs in a CCR5-dependent manner by secreting CCL4 and CCL5 (12). In addition, to further increase the complexity of the immunosuppressive network, Tregs have been identified as an important source of IL-10 in the TME. High IL-10 production levels amplify the immunosuppressive mechanisms sustaining the expression of FoxP3, TGF-*β*R, and TGF-*β*. TGF-*β* plays a complex role in BC progression, since it acts as a tumor-suppressor in normal and premalignant cells and as a tumor promoter during the more advanced phases of tumor development, with several epigenetic modification of its signaling partners and target genes controlling this dual role (110). Indeed, while under physiological conditions TGF-β inhibits mammary ductal growth and epithelial stem cell self-renewal, when released in the TME it induces EMT and the secretion of matrix components that stimulate invasion and metastatic spreading, and, together with VEGF, recruits endothelial cells and promotes their proliferation, favoring angiogenesis (111). Moreover, TGF-*β* participates in the downregulation of IL-2 expression, which is a requirement for T cell proliferation (44). Concomitantly, TGF-*β* favors the Treg infiltration in tumor tissues, which could also be directly induced by cancer cells through the expression of several chemokines, such as S1P, CXCL12, CCL20, CCL5, CCL28, and CCL2/22 (44).

As reported above, also TAMs, mainly as pro-tumoral M2, are abundant in the BCIM. TAMs originate primarily from bone marrow-derived blood monocytes/M-MDSC recruited in the TME and induced to rapidly differentiate into macrophages (12). Moreover, one of the main mechanisms found in different types of cancer, including BC, is the secretion of CCL2 through which the cancer cells are able to attract and increase the TAM abundance into the TME (112, 113). The presence of TAMs has been associated with the secretion of an array of chemokines, cytokines, and enzymes able to induce immunosuppression and to downregulate the activation of immune cells involved in the anti-tumor response. Notably, chemokines such as CCL2, CCL5, CCL17, CCL18, CCL20 and CCL22, cytokines such as hepatocyte growth factor (HGF), PDGF-B, VEGF, IL-4, IL-10, prostaglandin (PG) and TGF-β and enzymes, such as Cathepsin K, cyclooxygenase-2 (COX-2), ARG1 and MMPs secreted by TAMs can directly inhibit both CD8^+^ and CD4^+^ T cell effector function as well as recruit Tregs into the tumor lesion (114). In particular, PGE2, the major product of COX-2, plays a pivotal role in BC progression, though the binding to seven transmembrane G-protein-coupled receptors expressed on several immune cell subsets (115). Inhibition of its production by unselective COX inhibitors such as aspirin or other non-steroidal anti-inflammatory drugs has been associated with a reduced risk of developing BC (116), which constitutively expresses high amounts of COX-2 (117). PGE2 is secreted by both cancer cells and immune cells present in the TME, where it promotes the differentiation of MDSCs, from bone marrow progenitors, and DCs and their recruitment and activation, the M2 polarization of macrophages and their expression of programmed death ligand (PD-L)1. In addition, it suppresses NK anti-metastatic activity by reducing the expression of their activating receptors, stimulates the induction of Th2 cells and Tregs while inhibiting Th1 polarization, overall inducing immunosuppression (115).

Besides chemokines, cytokines, and eicosanoids, an immunosuppressive role is also played by metabolites produced by cancer cells, such as adenosine. Adenosine is a purine nucleoside present at low levels in healthy tissues, but released in high amounts in inflamed tissues and in the TME, where it acts as a danger signal. Adenosine is produced by the ectoenzyme CD73 from AMP, generated by CD39 starting from ATP. These two ectoenzymes are expressed at high levels on MDSCs and tumor cells from different cancers, and correlate with poor response to therapy in TNBC patients (118). Adenosine binds four different G-protein-coupled receptors that have been found to be expressed on multiple immune subsets. It exerts several immunosuppressive activities, such as the inhibition of activation and proliferation of CD4^+^ T and NK cells, induction of Tregs, skewing of DCs to tolerogenic or regulatory subsets and of macrophages to the M2 phenotype (61, 118) (**Figure 2A**).

### Cell–Cell Contact-Mediated Immunosuppressive Mechanisms

An additional strategy by which BC cells are able to evade immune destruction is mediated by cell-cell contact. Plasma membrane receptors such as the Programmed Death 1 (PD-1) and the Cytotoxic T lymphocyte antigen 4 (CTLA-4) are responsible for the T cell anti-tumor suppression activity, leading to tumor escape from the immune surveillance (119). In normal conditions, PD-1 is expressed on T and B lymphocytes, providing peripheral tolerance and protection against autoimmunity, while its ligand PD-L1 is mainly expressed on the surface of antigen-presenting cells. In pathological conditions, such as cancer, the cells can acquire the capability to overexpress PD-L1 and PD-L2. Although the mechanism is not completely understood, the PD-1/PD-L1/PD-L2 axis is able to induce anergy and/or apoptosis of PD-1^+^ T cells, attenuating the anti-tumor immune response and promoting Treg immunosuppressive activity (120, 121). Interestingly, a higher PD-L1 expression has been observed in HER2^+^ BC and TNBC subtypes rather than in the Luminal subtypes (122, 123).

In addition, CTLA-4, which belongs to the immunoglobulin superfamily, is expressed mainly on activated T cells, playing the role of T cells activity inhibitor. In fact, CTLA4 is homologous to CD28, a T-cell co-stimulatory protein, able to bind CD80 and CD86 on antigen-presenting cells. Thanks to its role in inhibiting the immune response against the tumor, CTLA-4 correlates with a poor prognosis in BC patients. Interestingly, besides on T cells, CTLA-4 is often expressed on BC cells (124, 125). Although its exact role in BC cells is still unknown, it might contribute to the regulation of PD-L1 expression and cell proliferation, as observed in lung cancer (126). Moreover, BC cells not only express these receptors on their surface, but they can also induce PD-1 expression in other immune cell populations, enhancing their immunosuppressive function. In particular, it has been described that tumor cells can modulate PD-L1 expression on MDSCs through the release of cytokines such as IFN*γ*. In fact, IFN*γ*-activated pSTAT1 is able to activate IRF1 protein, leading to its binding on a specific sequence in the *cd274* promoter, enhancing PD-L1 transcription. In fact, IFN*γ* is highly expressed in cells of the tumor tissues and its neutralization significantly decreased PD-L1^+^ MDSCs in the TME *in vivo* (127).

Furthermore, previous works demonstrated that also Tregs, accumulated in BC microenvironment, express high levels of CTLA-4 and PD-1, participating in T cell inhibition (128).

Interestingly, in addition to PD-L1 and CTLA-4, BC cells often upregulate other immune checkpoint (IC) markers as a mechanism of resistance to current inhibitors (129). For instance, T-cell Immunoglobulin and Mucin domain-containing molecule 3 (TIM-3) correlates with the presence of other IC markers such as lymphocyte activation gene (LAG)-3 and PD-L1 (129). TIM-3 is an IC receptor that is emerging as a target for cancer immunotherapy. It is expressed on both tumor and immune cells, and contributes to immune tolerance (130). LAG-3 is a cellular receptor expressed by activated T lymphocytes and is associated with T cell exhaustion (131), and it is commonly upregulated with PD-1 (132). Additionally, the T cell immunoglobulin and ITIM domain (TIGIT) co-inhibitory receptor (131), is highly expressed on CD8^+^ and CD4^+^ TILs in TNBC, while its ligands are present on antigen presenting cells and cancer cells (133). These three ICs, due to their properties, have been proposed as prognostic markers in BC, together with CD47 (131, 132, 134, 135). The CD47 receptor is expressed on the surface of several types of cancer cells and functions as an anti-engulfment signal that protects cells from phagocytosis by macrophages (136, 137). In particular, it is highly expressed on TNBC, and it has been associated with EMT and poor prognosis (135) (**Figure 2B**).

### Exosomes and Microvesicles as Important Players in Sustaining Tumor Progression

Due to their lipid double layer, extracellular vesicles (EVs) are able to carry stably active biological molecules and have a crucial role in cellular communication and trafficking in both physiological and pathological conditions. Exosomes are a subclass of EVs involved in intercellular communication that are released by all cell types, including cancer cells. Cancer exosomes have been demonstrated to mediate the main steps of tumor progression, in particular through the modulation of immune response, TME reprogramming and metastasis formation (138). It has been reported that BC cells often release exosomes containing TGF-*β* and IL-10, leading to T cells suppression (139–141). In particular, it has been shown that tumor-derived EVs are predominantly taken up by MDSCs, inducing MDSC immunosuppressive functions (142).

Moreover, it has been shown that tumor-derived exosomes could carry PD-L1 on their membrane surface. Besides inhibiting effector T cell recruitment and activation, exosome PD-L1 confers resistance to ICI therapy. Their ability to competitively bind to PD-L1 antibodies may contribute to the still largely unknown mechanisms of resistance of exosomal PD-L1 (143).

Numerous studies have underlined the role of exosomes in processes involved in tumor progression and survival, modulating immune cells such as DCs, T cells, macrophages, and NK cells and exerting a pro-inflammatory effect (144). For example, BC-derived exosomes can induce a pro-inflammatory response in macrophages localized at distant sites through the activation of NF-*κ*B, which in turn stimulates production of inflammatory cytokines (145). In particular, palmitoylated proteins on the cancer exosome surface are able to bind to TLR2 enhancing NF-*κ*B activation. In turn, activated macrophages prepare pre-metastatic niches that favor colonization by tumor cells (145). Furthermore, despite the molecular mechanism is not fully understood, it has been shown that tumor-derived EVs are able to increase the expansion of CD4^+^CD25^+^FoxP3^+^ Treg cells, inducing their suppressor activity and at the same time blocking the proliferation of activated CD8^+^ T cells (141, 146).

Interestingly, it has been demonstrated that BC-derived exosomes can contain and transmit also non-coding RNA, such as lncRNA SNHG16, which is able to induce CD73 in *γδ*1 Treg cells, enhancing their immunosuppressive effect *via* adenosine generation (147). Further studies have underlined the presence in EVs of miRNAs able to contribute to tumor progression. For instance, BC-secreted exosomal miR-105 could induce a metabolic program in cancer associated fibroblasts by activating the MYC signaling, adapting them to a different metabolic environment (148, 149). Another example is miR-503 that can enhance polarization of the microglia from a tumor-suppressive M1 to a tumor-promoting M2 phenotype, thus contributing to brain metastasis in BC (150). Interestingly, hypoxic conditions favor the release of immunosuppressive exosomes by BC cells. In fact, hypoxia increases the EV content of two immunosuppressive factors, TGF-β1 and miR-23a, which inhibit NK cell function by directly targeting the expression of CD107a and decreasing the cell surface expression of the activating receptor NKG2D (151) (**Figure 2C**).

## Importance of the TME in Response and Resistance to Immunotherapy

### Immunotherapy in BC

Immunotherapy has entered the clinical practice for BC patients as early as 1998, with the FDA-approval of the humanized HER2 monoclonal antibody trastuzumab, followed by other HER2 targeting antibodies (152). These drugs improved overall survival of patients affected by early or advanced HER2^+^ BC. However, tumors often display intrinsic or acquired resistance mechanisms, and most patients eventually experience disease progression (153).

Besides these passive immunotherapies, active immunotherapy for BC has been extensively studied. Although encouraging results came from preclinical analysis, most of the clinical trials with vaccines targeting tumor associated antigens (TAA) such as HER2 or mucin (MUC)1 failed to significantly improve patients’ outcome (154). Currently, new vaccines based on tumor-specific neo-antigens and shared oncoantigens that play a key role in the biology of CSCs are giving promising results that will hopefully pave the way for their clinical translation (155–159). Recently, immunotherapy options for BC treatments have expanded, with the introduction of the ICI atezolizumab (a PD-L1 antibody) in combination with chemotherapy for the treatment of patients with PD-L1^+^ unresectable locally advanced or metastatic TNBC (152). However, the Phase III double-blind IMpassion130 trial (ClinicalTrials.gov NCT02425891) demonstrated a clinically meaningful but not statistically significant difference in OS between patients treated with nab-paclitaxel plus atezolizumab or placebo, and a complete response rate of only 10.3% in PD-L1+ patients subjected to the combinatory treatment (160, 161).

Altogether, the results coming from the different BC immunotherapy regimens applied so far either in the clinical practice or in clinical trials suggest that multiple tumor cell intrinsic and extrinsic mechanisms of resistance need to be targeted to increase their efficacy. In particular, it is becoming increasingly evident that the immunosuppressive activity of the TME greatly affects tumor response to immunotherapy (5).

### The Role of TME in the Response to HER2-Targeted Therapies

The composition of the TME is key in determining the sensitivity of HER2^+^ BCs to HER2-targeted therapies (153). Indeed, several studies have shown that the presence of TILs and the expression of immune-associated gene signatures in pre-treatment biopsies are associated with longer DFS in HER2^+^ BC patients treated with anti-HER2-based therapy in the neoadjuvant or adjuvant settings (50). This is mainly due to the ability of immune cells to enhance trastuzumab anti-cancer activity. In fact, NK-dependent antibody-dependent cellular cytotoxicity (ADCC) plays a central role in trastuzumab-mediated cancer cell killing (162). Moreover, trastuzumab-induced HER2 internalization leads to HER2 presentation in MHC class I molecules, which can activate anti-tumor CD8^+^ T cells (163). Therefore, although the main mechanisms responsible for primary or acquired resistance to trastuzumab and to the other HER2-targeted therapies are cancer cell-intrinsic (153), the presence of M2 macrophages and other immunosuppressive cells in the TME significantly impairs the efficacy of HER2-targeting antibodies (164). Interestingly, trastuzumab treatment can increase the immune evasive properties of BC cells through the induced secretion of TGF-*β*, IL-6 and other immunosuppressive cytokines that, in turn, recruit immunosuppressive cells (165, 166). Indeed, the presence of a high number of TILs in patients with residual disease after neoadjuvant therapy was associated with worse DFS (167), probably due to an increase in Treg cells (168), indicating that immune-mediated resistance mechanisms need to be inhibited in BC patients to guarantee a good response to HER2-targeted therapy.

### TME Mediates Resistance to Immune Checkpoint Inhibitors

The poor response of most BCs to single-agent ICI therapy reflects intrinsic or acquired resistance (169). The mechanisms responsible for acquired resistance to ICIs in BC are currently unclear. However, lack of TILs and the presence of high numbers of MDSCs and other immunosuppressive cells correlate with low response (170). Of note, the TME composition in primary cancer differs from that in metastases, and clinical and preclinical data have demonstrated that primary BC are more responsive to ICIs than their corresponding lung or liver metastases, demonstrating that TME is important in determining response to immunotherapy (171). Indeed, many cells within the TME can impair the response to ICIs by inhibiting effector T cells (172), and depletion of intra-tumor MDSCs or Treg cells improved responsiveness to PD-1/PD-L1 blockade in preclinical models of BC (173, 174). Therefore, association of ICIs with therapies that revert the immunosuppressive activity of TME may improve their efficacy.

## Strategies to Revert Immune Suppression and Improve Cancer Immunotherapy

**Figure 3:**
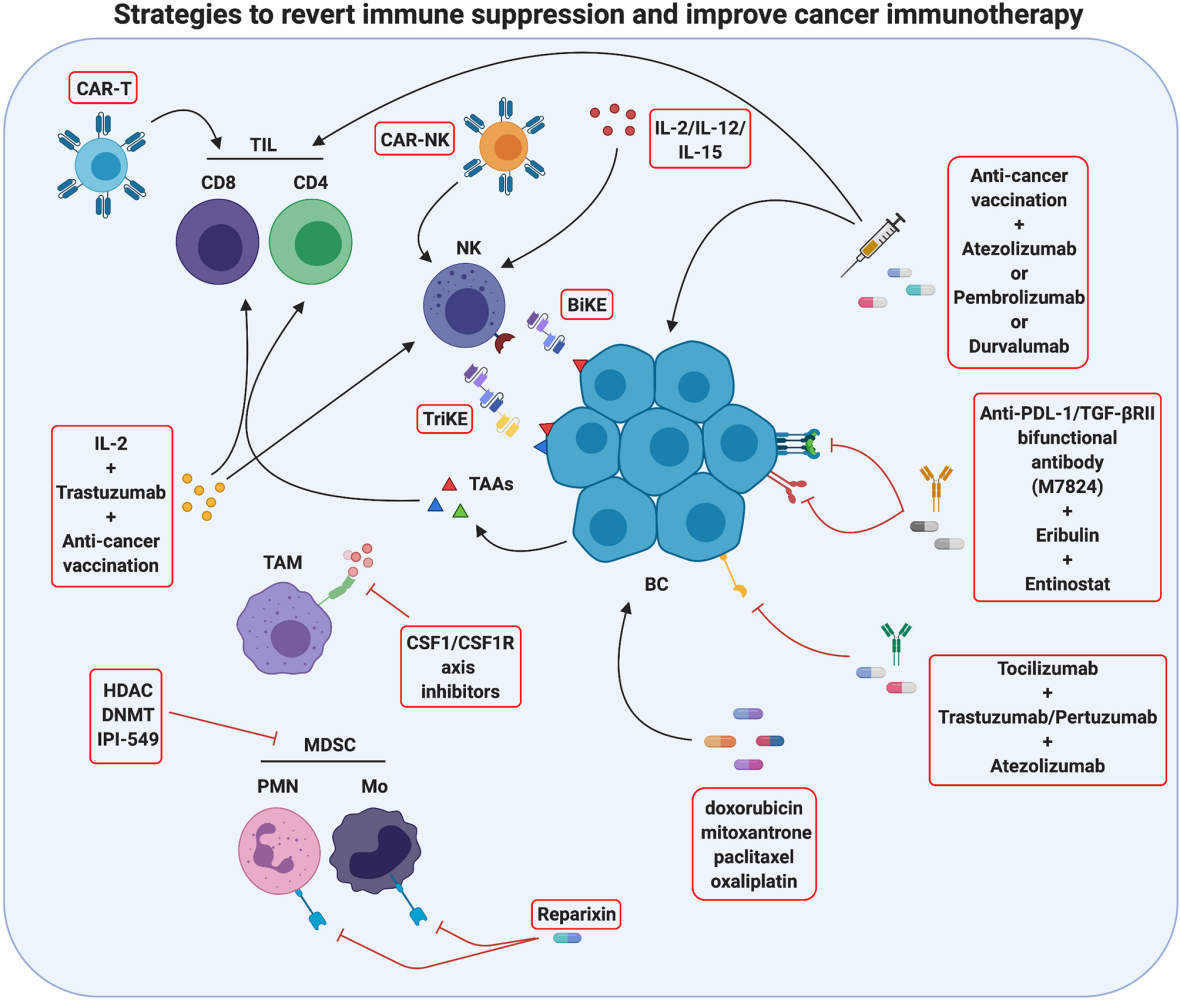
Strategies to revert immune suppression and improve cancer immunotherapy. Actually, several methods used in breast cancer treatment take advantage of immunomodulation mechanisms and are promising tools for tumor immunotherapy. One strategy is to improve immune cell activity against the tumor by innovative therapies such as CAR-T/CAR-NK administration, which employs patient’s T/NK cells engineered with chimeric receptors targeting antigens characteristics of cancer cells, or as anti-cancer vaccination, which stimulates the activation of the patient’s tumor-specific T cells. An additional method ongoing in clinical studies is the use of monoclonal antibodies against immune checkpoints or immune checkpoints inhibitors (ICIs) to block T-cell suppression, and the use of BiKE and TriKE reagents to induce antitumor NK cell activation. Moreover, an antitumor immune response may be activated by chemotherapy drugs inducing immunogenic cell death. A second strategy to improve tumor immunotherapy is to revert the immunosuppressive activity of TME often displayed in several breast cancers. In this sense, cytokine pharmacological modulation or inhibition of specific immunosuppressive pathways can be performed. The effects of single or combined therapies are actually studied. Created with BioRender.com.

The growing understanding of the mechanisms that cause resistance to immunotherapy will pave the way to the development of combination strategies that associate immunotherapy with drugs able to revert TME immunosuppression. Indeed, several studies have demonstrated that therapies that either recruit T or NK cells or reduce immunosuppressive factors in the TME can sensitize poorly immunogenic tumors to immunotherapy (175) (**Figure 3**).

The first strategy to improve the effectiveness of immunotherapy, and in particular of ICIs, is to recruit and activate effector cells such as anti-tumor T lymphocytes, since ICIs are not able to unleash antitumor responses if fully primed T cells are not present at the tumor site (176). This effect may be obtained with adoptive cell transfer therapy, and in particular with the administration of chimeric antigen receptor (CAR) T cells. Several clinical trials with CAR T cells specific for different tumor antigens such as HER2 (NCT03696030), EpCAM (NCT02915445), MUC1 (NCT04020575 and NCT02587689) and mesothelin (NCT02792114), alone or in combination regimens, are currently ongoing in BC patients. Till now, CAR T efficacy in solid tumors has demonstrated limited, mostly due to the presence of physical barriers that limit their infiltration in the tumor and to the immunosuppression exerted by the TME, but their combination with ICIs and other immunotherapies is expected to ameliorate cancer patient outcomes (177). However, it must be taken into account that, due to the difficulties in finding BC specific antigens, CAR T cells have been generated that targets TAAs, and therefore they can induce cytokine release syndrome and other severe reactions (178), as observed in a patient who died of pulmonary distress 5 days after receiving HER2-targeting CAR T cells (179), rising safety concerns.

Anti-cancer vaccination is a promising alternative to induce T cell recruitment in the tumor. Recently, cancer vaccines have been repositioned as a way to activate an immune response whose brakes are then removed by ICIs (154). A phase I clinical trial is testing the association of the personalized cancer vaccine RO7198457—an mRNA-based vaccine targeting an unspecified amount of tumor-associated antigens expressed in the patient’s tumor—with atezolizumab in patients with TNBC and other solid tumors (NCT03289962). Moreover, several clinical trials are currently recruiting patients affected by TNBC or other advanced BCs that will be treated with vaccination in association with the anti-PD-1 pembrolizumab or the anti-PD-L1 durvalumab (NCT04024800; NCT03362060; NCT03632941; NCT03789097; NCT04634747; NCT04418219; NCT03199040; NCT03606967; NCT02643303)). In the next years, the results coming from these trials will clarify the effectiveness of combined therapies based on ICIs and vaccination for BC treatment. Interestingly, cancer vaccines targeting CSCs can also synergize with HER2-targeted immunotherapy, as we have recently demonstrated in a preclinical model (180). Thus, multiple combination strategies might be developed in the next years to further improve BC treatment.

Another strategy to induce T cell activation and increase the efficacy of immunotherapy in BC patients is its association with cytotoxic chemotherapies that can induce immunogenic cell death (ICD), with the subsequent release of tumor antigens that prime T cells. Not all cytotoxic agents lead to ICD, but doxorubicin, mitoxantrone, paclitaxel and oxaliplatin do (181, 182). Moreover, these immunomodulating drugs improve immunotherapy by downregulating PD-L2 and upregulating MHC class I expression on tumor cells, increasing their immunogenicity (183). Several clinical trials testing the combination of ICIs with immunogenic chemotherapy have been performed (some examples are NCT02555657; NCT02622074; NCT03139851; NCT02425891), and the results from the IMpassion130 trial (NCT02425891) have led to the FDA approval of atezolizumab in association with nab-paclitaxel for first line, metastatic, PD-L1^+^  TNBC (160, 161).

Very recent data have shown that ICIs can act not only on T cells but also on NK cells, which express several ICs that inhibit their cytotoxic function, such as PD-1, TIM3, TIGIT, LAG-3 and CD96 (184). Several clinical trials are ongoing investigating the effects of ICIs on NK cells in different solid cancers, as reviewed in (185). Besides the classical ICs, NK cells express specific inhibitory receptors such as KIRs and NKG2A, and several inhibitory receptor blocking antibodies are currently undergoing clinical evaluation in solid cancers. Monalizumab, a mAb targeting NKG2A, is currently being tested in combination with trastuzumab in metastatic HER2^+^ breast cancer (NCT04307329). However, the study of these novel drugs in BC patients is still limited. Nevertheless, since NK cells represent an attractive tool for cancer immunotherapy thanks to their ability to kill cancer cells in an MHC-independent manner, other NK-based immunotherapies have been developed (186). Besides stimulation with cytokines (such as IL-12, IL-15 or IL-2, discussed below), the anti-tumor effects of endogenous NK cells can be stimulated by administration of bispecific and trispecific killer cell engagers (BiKE and TriKE, respectively), constituted by antibodies targeting CD16 or NKG2D and one or two tumor antigens (61). TriKEs can also be engineered to sustain NK cell proliferation *in vivo*, through the insertion of a modified IL-15 cross-linker (51). BiKe and TriKE specific for HER2 or EpCam were developed for BC, and, during the revision of this paper, GT Biopharma announced the initiation of clinical development of TriKE therapy for the treatment of HER2^+^ breast and gastrointestinal cancers, using a tri-specific scFv recombinant fusion protein conjugate composed of anti-CD16 and anti-HER2 antibodies, and a modified form of IL-15 (61).

Recently, adoptive NK cell therapy strategies have been explored in preclinical and clinical studies. Although adoptive transfer of autologous NK cells expanded *ex vivo* induced only very limited antitumor effect in patients with solid cancers, partially due to the immunosuppressed state of patients’ NK cells, a phase I clinical trial in patients with treatment-refractory HER2^+^ solid cancers treated with trastuzumab, bevacizumab, and autologous *in vitro* expanded NK cells reported preliminary antitumor activity, supporting the assessment of this approach in phase II trials (187). Alloreactive human pluripotent stem cell- or PBMC-derived NK cells have been widely investigated. However, in BC patients a phase II trial with allogeneic NK cell administration after lymphodepleting chemotherapy and total body irradiation gave poor results (188).

The difficulties in obtaining large amounts of NK cells led to the development of NK cell lines, among which EBV-transfected NK-92 is the only approved by the FDA for use in clinical trials (189). A clinical trial associating the infusion of NK-92 cells to the IL-15 super-agonist N-803 that promotes enhanced NK cell function, several chemotherapeutic drugs and vaccines targeting CEA, Ras and MUC-1, is currently recruiting TNBC patients who have progressed on standard of care therapy (NCT03387085). In order to improve their efficacy, NK cells expressing tumor-targeting CARs were generated. Autologous, allogeneic and NK cell lines can all be engineered to express CARs. Most CAR-NKs developed so far were tested in hematological malignancies, and some clinical trials are currently evaluating the safety and efficacy of PD-L1 or HER2-targeting CAR-NK therapy in solid tumors, although, to the best of our knowledge, there is not published data on human trials on BC up to now (186). Of note, the identification of CD142 (also known as tissue factor) as an antigen highly expressed in TNBC cells and CSCs, led to development of CAR-NKs specific for this aggressive type of BC, which led to positive results in preclinical studies (190), and similar results were obtained with EGFR-CAR NK cells (191), opening the way for a clinical development. Although CAR-NK therapy is still under evaluation, it displays potential advantages over CAR-T cell therapy. Indeed, NK cells release mainly IFN*γ* and GM-CSF, which are relatively safer than the cytokines released by activated CAR-T cells (IL-6 and TNF-α) that can cause cytokine release syndrome. Finally, CAR-NK cells can kill target cells in both CAR-dependent and CAR-independent manners, increasing their efficacy (51).

Another strategy to improve tumor immunotherapy is to revert the immunosuppressive activity of TME that characterizes most BCs. To this end, both drugs that deplete immunosuppressive cells and inhibitors of inflammatory cytokines have been tested. No selective MDSC inhibitors are currently known; however, many existing drugs reduce systemic and intratumor MDSCs, potentiating immunotherapy over time (15). DNA methyl transferase (DNMT) and histone deacetylase (HDAC) inhibitors, besides increasing tumor cell intrinsic immunogenicity through the upregulation of MHC class I and the antigen processing machinery (192), exert this effect (193). The HDAC inhibitor romidepsin is being evaluated in association with nivolumab and cisplatin in TNBC (NCT02393794), while the DNMT inhibitor decitabine in combination with pembrolizumab, followed by standard neoadjuvant chemotherapy, is under evaluation for locally advanced HER2^−^ BC (NCT02957968). Recently, a key role of PI3Kδ and PI3Kγ isoforms in promoting integrin4-dependent MDSC recruitment in the TME and in stimulating the immunosuppressive polarization of MDSCs and TAMs has been shown (194). Therefore, the PI3K*δ* and PI3K*γ* inhibitor IPI-549 is under evaluation in combination with atezolizumab and nab-paclitaxel in TNBC patients (NCT03961698).

Since in BC the dominant TAM phenotype is that of tumor promoting M2, which is associated with poor prognosis (195), macrophage depletion or re-education to anti-tumor M1 is an attractive approach for TME modulation (196). The most widely used strategy to date has been TAM depletion from the TME through inhibition of CSF-1/CSF-1R axis. CSF-1/CSF-1R inhibitors have been administered either as a monotherapy (NCT02265536) or in association with chemotherapy (NCT01596751 and NCT02435680). However, the available results from other cancer types showed only modest efficacy (196). This could be partially due to the ability of chemotherapy to recruit Tie^+^ macrophages in the TME, which in turn promote cancer cell dissemination (197). Therefore, a phase I clinical trial that evaluates the efficacy of the Tie2 kinase inhibitor rebastinib in combination with paclitaxel and the microtubule inhibitor eribulin mesylate in patients with advanced BC is currently ongoing (NCT02824575).

Aberrant overexpression of many proinflammatory cytokines has been reported in breast tumors, with a different profile during cancer progression (108). The modulation of cytokines present in the TME can be pharmacologically performed in order to either increase cytokines that promote anti-tumor immune responses or inhibit those that favor tumor progression (198). Among the anti-tumoral cytokines, IL-2 is one of the most studied, since it potentiates the activation of both cytotoxic T and NK cells, and can therefore enhance ADCC (198). IL-2 (aldesleukin or its pegylated more stable form bempegaldesleukin) administration has therefore been associated with trastuzumab, cancer vaccines or ICIs in several clinical trials in BC patients. However, the few results available so far indicate only a modest benefit (NCT00784524; NCT00003199; NCT03435640). This could be ascribed to the induction of compensatory immunosuppressive mechanisms, such as increased expression of IC molecules, secretion of inhibitory cytokines such as IL-10 and TGF-*β*, triggering of Treg cells and MDSCs, and activation of intracellular suppressors of cytokine signaling proteins that terminate the antitumor response (198). Therefore, many strategies that inhibit immunosuppressive cytokines have been developed and tested in a multitude of clinical trials in BC patients. TGF-*β*, IL-6 and IL-8 are among the most promising cytokines to be targeted, since their overexpression has been associated with advanced disease, higher risk of recurrence, stemness, therapeutic resistance as well as immune suppression (199–202). Several TGF-*β* targeting agents are under analysis in BC patients. An anti-PD-L1/TGF-βRII bifunctional antibody (M7824) is currently undergoing clinical evaluation either as a single agent in stage II–III HER2^+^ BC (NCT03620201) or in combination with radiation (NCT03524170), with eribulin (NCT03579472) or with a brachyury-targeting virus-based vaccine plus trastuzumab emtansine or the class I HDAC inhibitor entinostat in TNBC patients (NCT04296942). Similarly, the selective TGF-βR1 inhibitor galunisertib is under evaluation in combination with paclitaxel in TNBC patients (NCT02672475). For what concerns IL-6, the neutralizing IL-6 receptor antibody tocilizumab—FDA-approved for the treatment of cytokine release syndrome in CAR T-treated patients—is emerging as a potential new therapeutic in BC. Two clinical trials are recruiting patients to test its administration in combination with either trastuzumab and pertuzumab in metastatic HER2^+^ BC or with atezolizumab and nab-paclitaxel in advanced TNBC patients (NCT03135171 and NCT03424005). In preclinical models of TNBC, IL-8 inhibition was shown to revert the mesenchymal phenotype, decrease MDSCs in the TME and enhance tumor cell killing by T and NK cells (202), providing the rational for combining IL-8 inhibitors with immunotherapy or chemotherapy. Reparixin, a small molecule inhibitor of the IL-8 receptors CXCR1 and CXCR2, has been tested in clinical trials (NCT01861054; NCT01861054) in HER2^−^ BC patients, reporting a 30% response rate in 27 patients and a decrease in the aldehyde dehydrogenase CSC marker in about 25% of patients (203). Besides cytokines, molecules involved in the production of the immunosuppressive metabolite adenosine represent promising targets for BC therapy. In this light, clinical trials are currently ongoing in TNBC and other solid tumors combining immunotherapy with pembrolizumab or atezolizumab and inhibitors of CD73 or adenosine receptors (CPI-006 and CPI-444, respectively; NCT03454451 and NCT02655822), although the results have not yet been published (204).

## Conclusions

The introduction of immunotherapy has revolutionized the treatment of several cancer types, shifting the focus from cytotoxic therapies toward treatments that boost anti-tumor immune responses. However, only a small percentage of patients affected by BC currently benefit from immunotherapy. Indeed, the clinical efficacy of immunotherapy is limited to a subset of patients, and secondary resistance often develops in responding patients, further constraining the possibility of immunotherapy of substantially improving the outcome of BC patients.

A plethora of mechanisms contribute to the low efficacy displayed by immunotherapy in general, and of ICIs in particular, when administered as a single agent in the majority of BC patients, and it is now well known that the TME plays a pivotal role in the resistance mechanisms. Indeed, tumor progression is strictly intertwined with modifications of its TME that promote cancer cell proliferation while inhibiting the effector functions of anti-tumor immune responses, generating an immunosuppressive microenvironment that finally results in tumor outgrowth and metastatic dissemination. This immunosuppressive milieu generated by the crosstalk between cancer cells and immune and stromal cells present in the TME significantly dampens the protective anti-tumor immune responses activated by immunotherapies, thus resulting in treatment failure. The awareness of the existence of these mechanisms has shed light on the need to develop combination therapies that support the effect of ICIs and other immunotherapies by either expanding the activation and recruitment of effector cells, such as T lymphocytes and NK cells, or by inhibiting immunosuppressive cells and soluble factors. Of note, recent evidence from the literature and the clinics is expanding the focus of immunotherapy from its traditional T cell-centric view to a broader vision. Indeed, others and we have previously suggested that the humoral response plays a key role in immunotherapy-induced anti-cancer responses (157, 205, 206). This is particularly important considering that CSCs from BC and many solid cancers downregulate antigen-processing and presentation, thus escaping T cell responses (155). For the same reason, NK cells are emerging as new potential allies in cancer immunotherapy. Hundreds of clinical trials are currently testing different combinations of drugs, sometimes obtaining encouraging results. However, we must be conscious that BC and its TME represent a very heterogeneous and dynamic system that changes over time as the result of a complex crosstalk between neoplastic cells, immune cells and cancer therapies. This implies that a deeper understanding of the role played by the innate and adaptive immune response in individual BCs, and the characterization of the TME features that mostly influence the efficacy of immunotherapy, are needed to develop more effective treatments able to simultaneously activate anti-tumor immune responses and hinder the mechanisms leading to tumor immune escape. To this end, the identification of new predictive biomarkers of response to ICIs and combined therapies, which could help to stratify patients and guide the therapeutic decision, is urgently needed. Many efforts to define an immune signature distinctive of BC patients that positively respond to immunotherapy have been made, but clear-cut data are still missing (122, 207). The identification of personalized biomarker profiles, although representing a demanding challenge, may represent in the next years an important tool that could improve the development of optimal personalized combination therapies able to significantly improve BC prognosis.

## Author Contributions

LC, VS, and GC searched for current literature on the topic and wrote the manuscript. FC, PD, and LC reviewed the manuscript and finalized it for publication. All authors contributed to the article and approved the submitted version.

## Funding

This work was supported by MUR (Ministero Università Ricerca, PRIN 2015 to PD), AIRC (Associazione Italiana Ricerca Cancro) to PD (IG 20107) and to FC (IG 21468), Compagnia San Paolo, Torino, Progetto DEFLECT to PD and to FC, Fondazione CRT 2020.1798 to PD, and from the University of Torino (ex 60%) (Torino, Italy).

## Conflict of Interest

The authors declare that the research was conducted in the absence of any commercial or financial relationships that could be construed as a potential conflict of interest.

## Abbreviations

ADCC, Antibody-Dependent Cellular Cytotoxicity; APCs, Antigen-Presenting Cells; ARG1, Arginase 1; BC, Breast Cancer; BCIM, Breast Cancer Immune Microenvironment; bFGF, basic Fibroblast Growth Factor; CAR, Chimeric Antigen Receptor; CCL, CC-chemokine ligand; COX-2, Cyclooxygenase-2; CRTH2, Chemoattractant-homologous Receptor expressed on Th2 cells; CSCs, Cancer Stem Cells; CSF-IR, Colony-Stimulating Factor-1 Receptor; CSFs, Colony-stimulating factors; CTLA-4, Cytotoxic T Lymphocyte Antigen 4; CXCL, C-X-C-chemokine Ligand; DCs, Dendritic Cells; DFS, Disease Free Survival; ECP, Eosinophil Cationic Protein; EDN, Eosinophil-Derived Neurotoxin; EMT, Epithelial–Mesenchymal Transition; EPX, Eosinophil Peroxide; Evs, Extracellular Vesicles; G−, Granulocyte; GM−, Granulocyte-macrophage; HMGB1, High-Mobility Group Box 1 protein; ICD, Immunogenic Cell Death; ICI, Immune Checkpoint Inhibitor; Ics, Immune Checkpoints; IFN, Interferon; IL, Interleukin; ILCs, Innate Lymphoid Cells; IMCs, Immature Myeloid Cells; LAG-3, Lymphocyte activation gene-3; M, macrophage; MBP, Major Basic Protein; MCs, Mast cells; MDSCs, Myeloid-Derived Suppressor Cells; M-DSCs, Monocytic MDSCs; MHC, Major Histocompatibility Complex; MMPs, Matrix metalloproteinase; MUC, Mucin; NGF, Nerve Growth Factor; NK, Natural Killer; NO, Nitric Oxide; OS, Overall Survival; PD-1, Programmed Death 1; PDGF, Platelet-Derived Growth Factor; PG, Prostaglandin; PGD2, Prostaglandin D2; PlGF, Placental Growth Factor; PMN-MDSCs, Polymorphonuclear MDSCs; PNT, Peroxynitrite; RNS, Reactive Nitrogen Species; ROS, Reactive Oxygen Species; SCF, Stem Cell Factor; TAAs, Tumor Associated Antigens; TAMs, Tumor Associated Macrophages; TCR, T Cell Receptor; TGFβ, Transforming Growth Factor-β; TIGIT, T cell immunoglobulin and ITIM domain; TILs, Tumor Infiltrating Lymphocytes; TIM-3, T-cell Immunoglobulin and Mucin domain-containing molecule 3; TME, Tumor Microenvironment; TNBC, TripleNegative Breast Cancer; Tregs, Regulatory T Cells, VEGF, Vascular Endothelial Growth Factor.
